# Victims of human trafficking and exploitation in the healthcare system: a retrospective study using a large multi-state dataset and ICD-10 codes

**DOI:** 10.3389/fpubh.2023.1243413

**Published:** 2023-09-28

**Authors:** Alexander Gutfraind, Kezban Yagci Sokat, Guido Muscioni, Sami Alahmadi, Jonathan Hudlow, Ronald Hershow, Beau Norgeot

**Affiliations:** ^1^Carelon Inc., Indianapolis, IN, United States; ^2^Department of Medicine, Loyola University Chicago School of Medicine, Maywood, IL, United States; ^3^Division of Epidemiology and Biostatistics, University of Illinois School of Public Health, Chicago, IL, United States; ^4^Marketing and Business Analytics, San Jose State University, San Jose, CA, United States; ^5^Georgetown University, Washington, DC, United States; ^6^Love Justice International, Lincoln, NE, United States

**Keywords:** human trafficking, sexual exploitation, labor exploitation, primary care, ICD-10, insurance claims

## Abstract

Trafficking and exploitation for sex or labor affects millions of persons worldwide. To improve healthcare for these patients, in late 2018 new ICD-10 medical diagnosis codes were implemented in the US. These 13 codes include diagnosis of adult and child sexual exploitation, adult and child labor exploitation, and history of exploitation. Here we report on a database search of a large US health insurer that contained approximately 47.1 million patients and 0.9 million provider organizations, not limited to large medical systems. We reported on any diagnosis with the new codes between 2018-09-01 and 2022-09-01. The dataset was found to contain 5,262 instances of the ICD-10 codes. Regression analysis of the codes found a 5.8% increase in the uptake of these codes per year, representing a decline relative to 6.7% annual increase in the data. The codes were used by 1,810 different providers (0.19% of total) for 2,793 patients. Of the patients, 1,248 were recently trafficked, while the remainder had a personal history of exploitation. Of the recent cases, 86% experienced sexual exploitation, 14% labor exploitation and 0.8% both types. These patients were predominantly female (83%) with a median age of 20 (interquartile range: 15–35). The patients were characterized by persistently high prevalence of mental health conditions (including anxiety: 21%, post-traumatic stress disorder: 20%, major depression: 18%), sexually-transmitted infections, and high utilization of the emergency department (ED). The patients’ first report of trafficking occurred most often outside of a hospital or emergency setting (55%), primarily during office and psychiatric visits.

## Introduction

The United Nations defines human trafficking (also called ‘trafficking in persons’) as the “recruitment, transportation, transfer, harboring or receipt of persons, by means of the threat or use of force … for the purpose of exploitation” (1) and there are estimated to be tens of millions of victims worldwide (2, 3). In the US, the National Human Trafficking Hotline reported 64,718 victims with high confidence for 2007–2020 (4), and an estimated 199,000 incidents of sexual exploitation of minors may occur each year (5, 6).

The vast array of physical, sexual, psychological and social problems faced by survivors of human trafficking make it an important public health concern (7, 8) and healthcare providers are often on the operational frontlines when interacting with patients who are being trafficked (9, 10). However, even in clinical contexts, these patients may not make their experience known for reasons that include, but are not limited to, language or cultural barriers, fear of criminal repercussions, fear of the trafficker, or distrust of the healthcare provider (11, 12), limiting our ability to understand their characteristics and medical needs.

One emerging source of data on trafficking is a set of diagnostic ICD-10 codes introduced in late 2018. These 13 codes include diagnosis of adult and child sexual exploitation, adult and child labor exploitation, and history of exploitation. The codes are intended to be used in medical records of suspected and confirmed cases of trafficking, or identify patients with a history of trafficking (13). The codes are expected to result in better coordination of care for this vulnerable population and enable new research (14, 15). While the new codes have been launched in the United States only, there have already been calls to expand their usage globally (13). As healthcare providers are one of the major groups caring for victims of exploitation, the new codes have the potential to provide an important new source of data about exploitation resulting from trafficking (14), and improve health outcomes for this group of patients.

Here, we aimed to characterize the uptake of the new ICD-10 codes using a comprehensive national dataset, determine in which care settings a diagnosis is typically established, and to describe the medical needs of these patients. Our literature review with Pubmed, Scopus, Web of Science, and Google Scholar shows that there have been few studies based on the new ICD-10 codes. The two largest previous studies utilizing these codes relied on 49 pediatric tertiary centers (16) and 48 healthcare organizations (17). Another study (18) interviewed providers and examined their awareness of human trafficking but did not quantify the characteristics of the trafficked population. Additionally, a small study (*n* = 23) reported on how the codes were used to better identify sexually exploited youth and young adults in Minnesota (19). Our study is much larger in size (0.9 million medical organizations and 4 years of data) and based on a dataset that includes all providers that claimed payment for their services, regardless of size or location. It captures many more care settings including smaller providers and providers located outside major urban medical systems. Therefore, we hypothesized that the population of trafficked patients we will see will be more representative of the overall trafficked population.

## Methods

We conducted a retrospective, descriptive analysis of Elevance Health Inc.’s claims database. Elevance Health is one of the largest health benefits companies in the United States, managing healthcare for 47.1 million patients. The Elevance population is broadly representative of the overall US population and includes beneficiaries of commercial or government-provided health insurance across the US. The data consists of administrative claims, that is, medical bills created by medical facilities, professionals, and pharmacies and submitted to payers. In the US, the format of claims data is standardized by law (HIPAA) and contains a listing of diagnoses, health services, and medications. The data includes care settings such as large medical systems, independent medical groups and individual providers, with 0.9 million provider organizations overall.

We performed retrospective longitudinal search and reported on any ICD-10 diagnosis of exploitation between 2018-09-01, one month before the official launch date, and 2022-09-01. After finding the claims containing the codes, we counted all patients receiving the codes. To characterize recent patients of exploitation, in the analysis we excluded patients who only had codes associated with exploitation in the past or were victims of abuse: Z62.813/Z91.42 (personal history of exploitation) and Y07.6 (multiple perpetrators of abuse). We then compiled the medical claims for each of these patients in the 12 months before and after the trafficking event, but not within 7 days of it (to avoid inadvertently including misdated care related to the trafficking event), and reported any diagnosis and procedures codes in these intervals.

Trend was identified using regression for the daily number of bills containing the new ICD-10 codes, and separately (to avoid collinearity) regression for the number of all bills received each day. Because each medical provider has a small independent probability of using the codes on a bill, a generalized linear regression model was used. To ensure that the trend analysis is not affected by the quick uptake at the introduction of the ICD-10 codes in late 2018, the model was fit to data from 2019-01-01 through 2022-09-01. Goodness of fit testing evaluated the Negative Binomial (NB), Poisson and Gaussian models, and linear and quadratic trends (NB with a linear trend was strongly preferred).

We accessed an anonymized copy of the Elevance claims repository using a SQL client using Python 3.7, *pandas* (20) and the *sqlalchemy* package (21). Regression was fitted using *statmodels* (22). For the study, the research group was provisioned with a limited view of the repository, with minimized access to clinical data. Birth dates were randomly obfuscated within a calendar year. All data was encrypted at rest and during transmission and accessed from secured devices.

## Results

The dataset contained 5,262 instances of the ICD-10 codes (Table 1).

**Table 1:** Frequency of use of the new exploitation and trafficking codes from 2018-09-01 through 2022-09-01.

ICD-10 code	First event	Count	#Patients	Description
T74.51	10/5/18	494	184	Adult forced sexual exploitation, confirmed
T74.52	10/11/18	562	154	Child sexual exploitation, confirmed
T74.61	12/7/18	60	42	Adult forced labor exploitation, confirmed
T74.62	2/28/19	20	3	Child forced labor exploitation, confirmed
T76.51	10/3/18	240	124	Adult forced sexual exploitation, suspected
T76.52	10/9/18	321	151	Child sexual exploitation, suspected
T76.61	6/29/19	15	14	Adult forced labor exploitation, suspected
T76.62	10/13/18	24	10	Child forced labor exploitation, suspected
Y07.6	10/3/18	1,062	895	Multiple perpetrators of maltreatment and neglect
Z04.81	10/3/18	759	520	Encounter for examination and observation of victim following forced sexual exploitation
Z04.82	10/7/18	164	102	Encounter for examination and observation of victim following forced labor exploitation
Z62.813	10/4/18	709	387	Personal history of forced labor or sexual exploitation in childhood
Z91.42	10/9/18	832	372	Personal history of forced labor or sexual exploitation

Total patients: 2,793. Patients with a probable recent event: 1,248 (i.e., based on any codes other than codes Y07.6, Z62.813, and Z91.42). Count gives unique bills.

**Figure 1:**
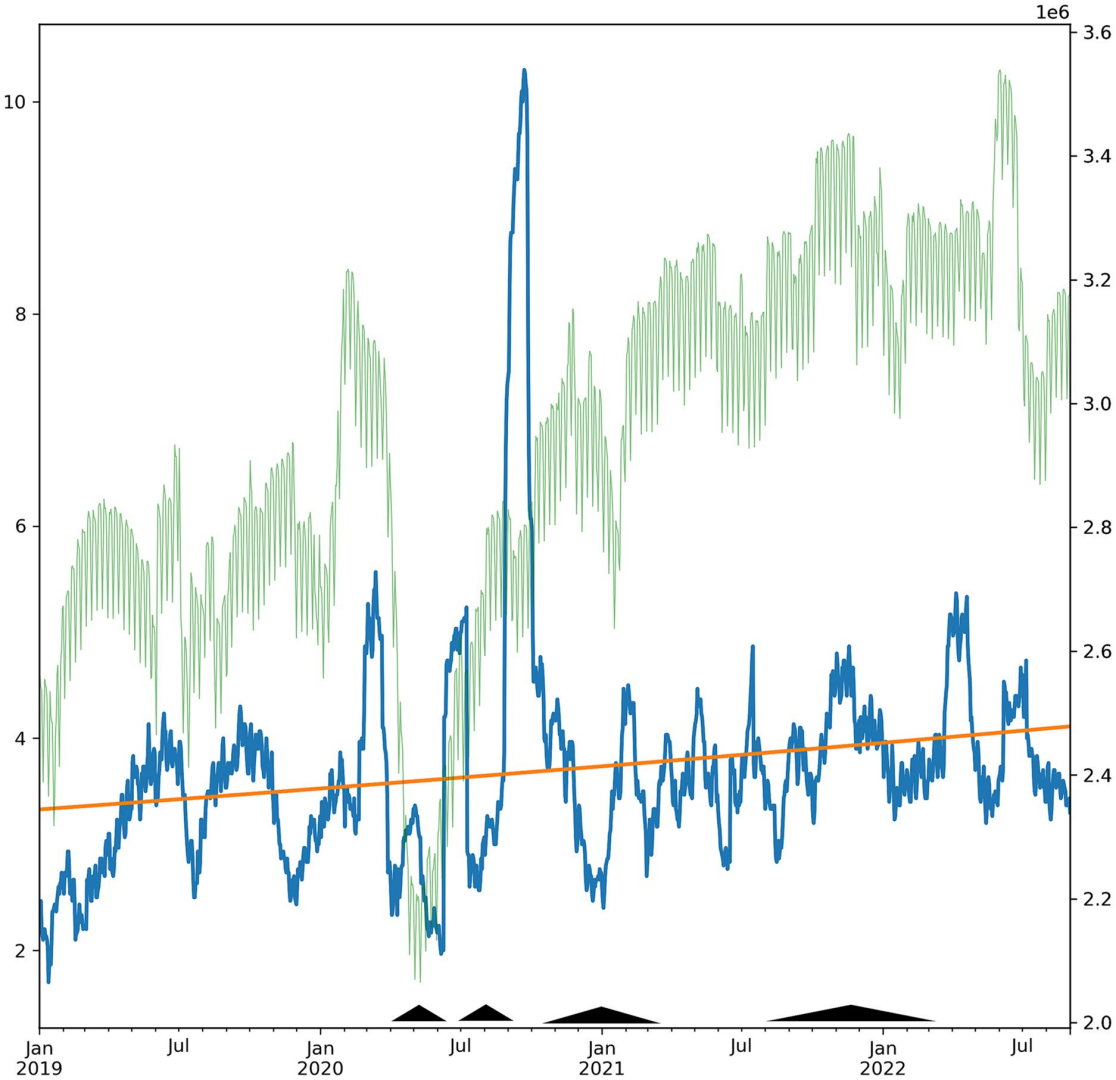
Daily count of exploitation-related ICD-10 codes from 2018-09-01 through 2022-09-01 (blue, left axis, 30-day moving average). Use of the codes increased 5.8%, representing a decrease of −0.9% per year relative to the growth of the data (6.7% per year). Superimposed trendline (orange) and overall new claims in the database (faint green, 30-day moving average). Pyramids indicate elevated in COVID-19 mortality in the US (periods with >5,000 weekly cases), as reported by the CDC COVID Tracker (covid.cdc.gov).

Regression analysis found a 5.8% (*p* < 0.05) increase in the adoption of these codes per year, representing a slower rate than the overall growth of the dataset (6.7% per year) (Figure 1).

The codes were used by 1,810 different medical providers (0.19% of total), with 77% reporting just a single patient. In 28% of the cases the codes were used as the principal or admitting diagnosis. The data contained 2,793 patients of which 1,248 were recently trafficked (reported on Table 2), while the remainder (55%) had a personal history of exploitation. Of the recently trafficked, 86% and 14% experienced sexual and labor exploitation, respectively, and 0.8% both types.

**Table 2:** Demographics of the population with new diagnoses of exploitation or trafficking (*n* = 1,025 patients).

	Typical value	Distribution information
Exploitation type	86% sexual	14% labor, 0.8% both types of exploitation
Age	20 years (median)	IQR: 15–35. Median sexual: 19.9, labor: 28.3 21% children under 15 and 52% under 25
Gender	82% Female	Fraction Female: Sexual: 83%, Labor: 8%
Race	60% Unknown	White: 49% White, 35% Black, 11% Latin-American and 3% Asian-American
Health insurance	63% Medicaid	18% large employers, 12% multi-state employers, and others (individual policies 0.4%)
	75% Primary subscriber	19% as child, 6% as spouse, 4% unknown/other (numbers do not sum to 100% due to rounding)

IQR, Interquartile range.

The patients were predominantly female (83%), insured by Medicaid (63%) and the median age was 20 (interquartile range: 15–35) with 21% children under 15 and 52% under 25. Race or ethnicity (as voluntarily self-reported during insurance enrollment) was 49% White, 35% Black, 11% Latin-American and 3% Asian-American.

Comparing the medical needs in the 12 months before and after the report of exploitation (Table 3) and Supplementary Table S1 found that the patients were characterized by persistently high prevalence of sexually-transmitted infections, mental health conditions (including anxiety: 21%, post-traumatic stress disorder: 20%, major depression: 18%), and high utilization of the emergency department (ED). Annual medical costs were high (mean $31,055 in the 12 months after diagnosis, standard deviation $152,442) compared to enrollees in Medicaid (mean $6,556 per year in 2019 – the last year with data) (23). The median cost was $5,254, suggesting that the costs were strongly skewed.

**Table 3:** Diagnosis codes among victims of exploitation.

(A) At establishment		(B) 12 month medical history	
Description and ICD-10 code	% Pts	Description and ICD-10 code	% Pts: 12 mo before/12 mo after
Encounter for examination and observation of victim following forced sexual exploitation (Z04.81)	42	Encounter for immunization (Z23)	29/27
Child sexual exploitation, suspected, initial encounter (T76.52XA)	11	Anxiety disorder, unspecified (F41.9)	21/21
Adult forced sexual exploitation, confirmed, initial encounter (T74.51XA)	11	Other long term (current) drug therapy (Z79)	21/20
Child sexual exploitation, confirmed, initial encounter (T74.52XA)	9	Major depressive disorder, single episode, unspecified (F32)	19/18
Adult forced sexual exploitation, suspected, initial encounter (T74.51XA)	9	Encounter for screening for sexually transmitted infection (Z11.3)	17/15
Encounter for examination and observation of victim following forced labor exploitation (Z04.82)	8	Post-traumatic stress disorder, unspecified (F43.10)	15/20
Post-traumatic stress disorder, unspecified (F43.10)	7	Unspecified abdominal pain (R10.9)	15/15
Encounter for immunization (Z23)	6	Suicidal ideations (R45.851)	15/12
Anxiety disorder, unspecified (F41.9)	6	Encounter for routine child health examination without abnormal findings (Z00.129)	15/14
Encounter for screening for sexually transmitted infection (Z11.3)	6	Cough (R05)	15/10
Suicidal ideations (R45.851)	5	Urinary tract infection, site not specified (N39.0)	14/11

(A) The most common diagnoses in encounters in which exploitation was first diagnosed, (B) The most common diagnoses over the preceding 12 months before the first exploitation diagnosis code, and their frequency 12 months before and after. % Pts: percent of patients with the diagnosis over the given period. Total patients: 1,248. If multiple related ICD-10 codes were used, the code reports the shared code prefix.

The first patient report of trafficking occurred most often outside of a hospital or emergency setting (55%): 25% during an office visit as established patients, 8% as new patients, 10% in psychiatric encounters, 4% in behavioral therapy, and the rest were diagnosed in other non-hospital settings. EDs accounted for just 25% of first reports.

## Discussion

In a national database, new ICD-10 codes usage reveals a population that has experienced and reported trafficking that is predominantly young and female. The population is affected by severe mental health conditions at rates much higher than the general population (5.6% in 2020) (24). The population was found to be slightly older and racially more White than reported in an earlier study by Garg and colleagues (16), likely because it utilized data from urban pediatric tertiary medical centers. COVID-19 likely interrupted the access of trafficking victims to their medical providers, as evidenced by the decline in diagnosis coinciding with peaks in COVID-19 mortality.

Generally speaking, it was surprising to see so many victims in our database, which includes just persons holding commercial or government-provided insurance, as it runs counter to the view that victims would have no insurance of any kind and receive care predominantly in emergency departments. Additionally, we were surprised to see slow uptake of the codes considering the large population of victims in the country, suggesting that there are significant barriers to adoption of the codes. While the overall volume of the data in our database grew due to increase in the enrolled population and increased medical utilization, growth in the usage of the codes was nearly 1% slower than the growth in the volume of data. Additionally, 35% of the codes indicated a suspected rather than the confirmed label suggesting that the providers were not able to obtain definitive evidence of trafficking or are in the process of investigating it. AAPC guidelines leave the label to the discretion of the medical provider.

Although the patients were often treated at emergency departments, in a break with earlier studies, the majority of the patients in our data were first diagnosed outside of hospitals as part of a routine visit as an established patient. A trusting and long-standing patient-physician relationship may enable patients to disclose a history of exploitation, which can lead to higher identification and reporting (25). Since such relationships are expected to appear more in primary care and psychiatric services compared to the emergency settings and our data is more comprehensive compared to other studies and includes smaller providers, it is not surprising to have the first diagnosis outside of the hospitals. The patients exhibited little or no change in their diagnoses when comparing 12 months before and after the diagnosis of exploitation, suggesting that they are unable to receive definitive care for their conditions or have chronic hard-to-treat conditions.

The available data, though large in scale, captures only a fraction of the trafficked patients nationally, and might disproportionately omit certain subgroups of patients. Underreporting might occur because patients might have barriers to accessing care, be concerned about shame, stigma, discrimination by staff or legal ramifications (26). Providers might lack training on the new codes or deliberately avoid their use for the fear of potential unintended consequences, patient privacy or wrongly labeling a patient. Providers might be more likely to suspect trafficking when the patient matches a certain profile, causing underreporting of patients that fall outside that profile. Additionally, patients and providers might be concerned about whether the trafficker has access to the medical records of the patient or not, which might make the providers hesitant about using the new ICD-10 codes (16). Because administrative claims data is used for billing, providers might sometimes be using more conventional diagnostic codes instead of trafficking, although they are incentivized to report as many medical conditions as possible. But we hypothesize that the lack of awareness and absence of institutional policies are the primary causes of underreporting, and therefore underreporting is highest in providers that see few trafficked patients. As a result, patients treated at emergency departments (EDs), youth medical centers and similar facilities are likely overrepresented in the data.

Another limitation is that the data includes only patients with medical insurance rather than those paying for care directly or receiving charitable care. The data does include patients with all types of health coverage, including employer-provided insurance, government-sponsored commercially-administered Medicaid and Medicare programs and individual policies. Because the health insurer is involved with patients over time and across providers, the data provides a comprehensive picture of the patients and their journeys over time. Patients without insurance likely have medical needs similar to our population but utilize medical services differently and might use emergency departments (EDs) more frequently than patients in our database. These populations are better captured in studies that rely on electronic medical record systems (16, 17). COVID-19 and measures taken in some states might have impacted the uptake of codes in those states (27), particularly, California and New York, which experienced lengthy shutdowns during part of the study period.

## Conclusion

Overall, our findings strengthen the case for increased vigilance for victims of trafficking in healthcare settings outside of hospitals. We agree with authorities (28–30) that recommended healthcare providers should receive targeted training in safe reporting of and caring for these patients. Such interventions could increase the uptake of the codes and improve care for the patients.

Future research should examine barriers for implementation of the new ICD-10 codes, and evaluate the perception that they could lead to stigmatization or loss of confidentiality. It would also be valuable to characterize qualitatively both the patient and provider perspectives on the use of the new codes, complementing the epidemiological analysis in this study. Lastly, more research should evaluate how COVID-19 and other medical crises affected or could affect victims of exploitation and the uptake of the new diagnosis codes.

## Data availability statement

The data analyzed in this study is subject to the following licenses/restrictions: The data are not publicly released due to HIPAA regulations and patient privacy. The corresponding author may be contacted to discuss data access following approved agreements. Requests to access these datasets should be directed to sasha.gutfraind@carelon.com.

## Ethics statement

The studies involving humans were approved by Elevance’s institutional review and ethics board. The studies were conducted in accordance with the local legislation and institutional requirements. The ethics committee/institutional review board waived the requirement of written informed consent for participation from the participants or the participants’ legal guardians/next of kin because the Board deemed the study a retrospective analysis for healthcare operations in accordance with HIPAA and 45 CFR 46.102(f), and waived the requirement for patient informed consent. All data utilized were de-identified and aggregated.

## Author contributions

AG led the design and implementation. KY conceived the study. GM contributed to the implementation. BN, RH, SA, and JH critically reviewed the manuscript and added important intellectual content. All authors contributed to the analysis and writing and agreed to be accountable for all aspects of the work.
